# Rapid assessment of insect fauna based on local knowledge: comparing ecological and ethnobiological methods

**DOI:** 10.1186/s13002-016-0085-z

**Published:** 2016-03-01

**Authors:** Daniele Cristina de Oliveira Lima, Marcelo Alves Ramos, Henrique Costa Hermenegildo da Silva, Angelo Giuseppe Chaves Alves

**Affiliations:** Graduate Program in Ethnobiology and Nature Conservation (PPGEtno), Federal Rural University of Pernambuco (UFRPE), Recife, Pernambuco Brazil; Department of Biological Sciences, University of Pernambuco (UPE), Campus Mata Norte, Nazaré da Mata, Pernambuco Brazil; Department of Biology Sciences, Federal University of Alagoas, Campus Arapiraca, Arapiraca, Alagoas Brazil; Department of Biological Sciences, Federal Rural University of Pernambuco (UFRPE), Recife, Pernambuco Brazil

**Keywords:** *Abelmoschus esculentus*, Local entomological knowledge, Semiarid environments, Ethnoentomology, Visual stimuli, Irrigated agriculture, Checklist interview, Okra

## Abstract

**Background:**

The rapid assessment of biodiversity making use of surveys of local knowledge has been successful for different biological taxa. However, there are no reports on the testing of such tools for sampling insect fauna. The present study aimed to evaluate the efficiency of different ethnobiological techniques for rapid sampling of insect fauna.

**Methods:**

Field research for the conventional survey of insect fauna was conducted on a private farm (9 ° 43'38.95 "S, 37 ° 45'11.97" W) , where there was intensive cultivation of okra (*Abelmoschus esculentus* L. (Moench)). The survey of local entomological knowledge was conducted among all the producers of okra living in the rural villages Pereira, Santa Luzia, and Nassau de Souza, within the Jacaré Curituba irrigated settlement scheme. The combined use of the techniques “free list” and projective interviews was analyzed, using two types of visual stimuli: stock photos and an entomological box.

**Results:**

During the conventional survey of insect fauna, the species *Bemisia tabaci* biotype B, *Aphis gossypii*, *Phenacoccus* sp., *Icerya purchasi* and *Lagria villosa* were the primary pests found in the okra crop. Regarding the survey of insect pests, the results were convergent  in both techniques (conventional sampling and free list). Comparing the interview with visual stimuli (pictures) and specimen witnesses (entomological box) revealed that the latter was more effective.

**Conclusion:**

Techniques based on the recording and analysis of local knowledge about insects are effective for quick sampling of pest insects, but ineffective in sampling predator insects. The utilization of collected insects, infested branches, or photos of the symptoms of damage caused by pests in projective interviews is recommended.

## Background

Knowledge of the diversity of insects associated with human populations is fundamental for ecological studies and pest management [1] and for programs monitoring the quality of environments, using insects as bioindicators [2]. Accordingly, the involvement of farmers is essential in such studies and programs, in that it saves time and money in surveying insect fauna [3].

The information accumulated over time by farmers in a mnemonic way [4] can be accessed through interviews, making it possible to create an inventory of known items within a category [5]. The literature reports that listings of organisms acquired through interviews point out especially the most culturally important items [6], considering that information remembered by people appears in order of familiarity [5].

Research involving farmers is an alternative to the inventories planned and conducted only by scientific professionals, which are slower and expensive [1–7]. Surveys of key pests can thus be done more quickly and less costly by interviewing farmers through rapid biodiversity assessment (RBA), contributing to development of monitoring protocols for insect fauna, which are scarce [8]. RBA is an approach that provides for the collection of environmental data in a short time, with the integration of multiple levels of information, possibly combined with data from local knowledge [9].

As for the use of data from local knowledge, ethnozoological studies have contributed to the enhancement of zoological research, on topics such as taxonomy and inventories [10] and ecological surveys and fauna distribution [11], hypotheses generation [12] or supplementing zoological inventories [13]. The integration of local and scientific knowledge may have a central role in animal conservation and management [10].

Silva et al. [9] pointed out the importance of involving parataxonomists (people capable of identifying biological samples without having had formal training in taxonomy and systematics) or local experts (persons recognized by the population as very knowledgeable about plants and/or animals in the region) in monitoring programs.

The RBA was created in the 1990s by the influence of the Convention on Biological Diversity (CBD) as a result of Rio 92. Since then, rapid assessment, making use of surveys of local knowledge, has been successful for different biological groups such as plants [9–14], mammals [15] and game animals [16, 17]. However, there are no reports that such tools have been tested for sampling insect fauna.

The RBA uses different techniques to survey local knowledge, such as focus groups [18], semi-structured interviews and free lists [17–19], participatory workshop and inventory interview [9], participatory rural appraisal and participatory mapping [20] guided tour [20] and interviews with visual stimuli [21]. An alternative incorporated into the RBA to inventory the insect fauna of a region could be a projective interview, which consists of using visual stimuli, such as cards, photographs, drawings, films, individuals in situ, dried specimens or live plants and animals, artifacts or products derived from plants or animals, at the time of interview, for information on local knowledge about a certain topic or elements of natural ecosystems [22–24]. It has been employed in the identification and recognition of natural elements [25], to acquire information about vernaculars and categories of uses of species [26] or to supplement information obtained in other research approaches [27, 28].

Thus, this study provides a methodological contribution to ethnobiological research by comparing different techniques for collecting data on agricultural insects, trying to answer the following questions. Would the insects most recognized by farmers be predominant in a conventional population survey of insect fauna? Can a survey of the most important pests of a crop (key pests) be performed using data from local knowledge? Among the most common visual stimuli for collecting ethnobiological data (photographs and dry specimens), which would be the most efficient for the recognition of insect fauna?

To answer these questions, we selected the okra crop (*Abelmoschus esculentus* (L.) Moench) as a model because of the importance of this vegetable crop currently in the regional economy and the lack of plant disease studies for this crop, especially in semi-arid Northeast region of Brazil. Over the past 10 years the municipality of  Canindé de São Francisco has been distinguished as the second largest okra producer in Brazil. Farmers in this municipality have favored the production of okra due to its adaptation to local environment and long uninterrupted harvest cycle [29].

This seems to be the first comparative approach on insect species as recognized by local farmers and external observers (scientists). In this study, we adopted the checklist-interview term for the technique that uses visual stimuli outside the original context of the animal (such as dried parts, fresh parts, photographs and sketches), as recommended by Medeiros et al. [26], who recognized the need for a methodological adjustment related to the use of visual stimuli in ethnoscientific research.

## Methods

### Study area

This research was carried out at Jacaré Curituba irrigation settlement scheme, located between the municipalities of Canindé do São Francisco and Poço Redondo. They are found in the in the Northwest of the State of Sergipe, Northeast Brazil, under the geographical coordinates of 08°29’23’S and 36°03’34”W. The settlement has been in operation since 1996 with a total area of 50.000 km^2^, organized in 38 rural villages, including irrigated sections and dryland (rain dependent) plots. The irrigated scheme is divided into 333 p lots with a total area of 39.800 km^2^, in which farmers use water collected directly from the São Francisco River.

Predominant soils in the irrigated area are Luvisols, eutrophic Lithosols, Vertisols, Cambisols and Alfisol [30]. The climate, according to Köppen, is Bssh, very hot, semiarid, steppe type, with rainy season centered in April, May and June [31, 32].

For 2013, period of data collection, the annual average rainfall rate in Canindé do São Francisco was 547.8 mm. The average air temperature was around 26 and 27 °C, with monthly minimum temperatures between 18 and 22 °C and monthly maximum temperatures ranging between 28 and 34 °C (Data obtained from the meteorological station located at the National Monument of Grota do Angico: 9°41’14.09”S,37°41’6.77”W).

The main crops grown by local farmers (also called “irrigators”) on the perimeter are okra (*Abelmoschus esculentus* (L.) Moench), bell pepper (*Capsicum annuum* L.), Antilles cherry (*Malpighia glabra* L.), banana (*Musa paradisiaca* L.), guava (*Psidium guajava* L.), mango (*Mangifera indica* L.), soursop (*Annona muricata* L.), pumpkin (*Cucurbita pepo* L.), cassava (*Manihot esculenta* Crantz), peanut (*Arachis hypogaea* L.), common bean (*Phaseolus vulgaris* L.), corn (Zea mays L.) and tomato (*Solanum lycopersicum* L.). Currently okra is one of the predominant crops in the Public irrigated settlement schemes in that portion of the São Francisco River watershed that belongs to the State of Sergipe [33].

### Conventional survey of insect fauna

The field research to survey the insect fauna was held at a private farm located in the Jacaré Curituba irrigated settlement scheme  (9°43’38.95”S; 37°45’11.97”W). Okra production in this farm follows mainly conventional methods, with periodic administration of synthetic fertilizers and insecticides.

Synthetic insecticides under usewere Engeo™ Pleno (Syngenta Proteção de Cultivos Ltda) and Lannate BR (Du Pont do Brasil S.A.). The insecticide Engeo™ Pleno was administered on July 24th and September 27th 2013, while Lannate BR was applied on 29th August, 30th October to 27th November 2013, respecting the recommendations of the product description leaflet. Direct sprays onto the leaves were performed using 20 l of backpack sprayer. The weed removal was done with weeding hoe. Besides okra, the coverage around the area was made up of cassava and corn crops.

An area of 72 × 76 m was used as a basis for the cultivation of okra, planted with the cultivar Santa Cruz 47, with a 30 cm spacing between rows. When the plants reached the reproductive phase, we monitored the density of nymphs and adults of mealybug *Phenacoccus* sp. and cotton aphids (non-winged) *Aphis gossypii* and *Icerya purchasi*, as well as eggs and nymphs of whitefly *Bemisia tabaci*. The monitoring of the latter was done by leaf collection technique in clear plastic bag. The adults of the other insects were collected with a vacuum cleaner (winged insects) or tweezers (wingless insects), placed in plastic bags and then labelled and fixed with entomological pins in the laboratory.

The leaf collection technique in clear plastic bag consisted in enwraping the leaf in a plastic bag, pulling it up, sealing it inside the bag, keeping in refrigerator (8–10 °C) and sending it forward to the laboratory, for determining the number of insects. The insects collected were assembled through entomological pins and kept in entomological boxes. Specimens were identified with the aid of identification keys or by comparison with the material deposited in the entomological collection of the Federal Rural University of Pernambuco.

In order to establish the sampling points, the field was divided into twelve transects, separated by 6 m each, used as guides to the collections. Two plants were sampled by transect, each inspected for 5–6 min per person.

The collections of phytophagous insects and predators were carried out fortnightly during the critical period of attack of pests reported by producers (August–November 2013), with a total of 9 samples in 120 days. All samples were performed in daytime, between 8 am and 12 pm.

### Ethnobiological survey of insect fauna

The first informant was indicated by the leader of the Jacaré Curituba irrigated settlement schem. From then on, the selection of informants proceeded with the application of the “snow ball” sampling technique [22], with the Informants being indicated by other residents who considered them to be knowledgeable about insects. The interviews were considered finished when the names indicated began to be repeated.

At this stage, 96 farmers were interviewed. Upon completion of the interviews by snowball sampling, 43 “local experts” were especially selected to evaluate the efficiency of different techniques for the rapid sampling of insect fauna. The local experts were individuals who were repeatedly pointed out certain insects during 96 initial interviews with randomly selected farmers [22]. The selected farmers were residents of the farming villages Pereira, Santa Luzia and Nassau de Souza. These farming villages have had irrigated cultivation of okra for the longest time in the Jacaré Curituba settlement (about 10 years). In these farming villages, plantations are irrigated by gravity with water from a reservoir, since before the implementation of the irrigation scheme by the Development Company of the Valleys of the São Francisco and Parnaíba (CODEVASF), a State Agency. Of the 43 selected farmers, 36 agreed to participate in the study (30 men and 6 women, aged 21–63 years). The majority (89 %) were from neighboring municipalities and worked in dryland farming since childhood. The difference in the number of men and women is related to the fact that irrigated agriculture remains locally a predominantly male practice.

Initially, research objectives were presented to farmers and local leaders at a meeting of the settlement. Once given verbal consent for the study to get started, each interviewee was asked to sign the Informed Consent, fullfiling the requirements of the National Health Council (Resolution No. 292 of 08/07/1999). This study was approved by the Ethics Committee of the University of Pernambuco (CAAE- Protocol 24844813.0.0000.5207).

The first phase of work consisted of the use of the ethnographic technique “free list” [22]. The objectives were to: a) obtain and analyze specific information about the cultural field investigated (the okra damage causative insect); b) calculate the consensus on the cultural items of all recognized insects; c) determine from the discourse of informants key pests and natural enemies for okra culture. In the free list technique respondents were asked to list, according to their discretion, all insects that occur in okra. From them, we included only organisms within the class Insecta. Some techniques were used during interviews to enrich the information provided, such as non-specific induction, new reading and semantic suggestion [22].

In another moment projective interviews were applied to 36 people who had been interviewed in the first phase. This technique is similar to the projective tests and checklist-interview method [26]. This one involves the application of visual stimuli to help obtaining information on local knowledge about a certain topic. It is sought, in this case, to identify the categories of insects that local producers recognize more frequently among those that had been found in the conventional sampling field.

The visual stimuli used in the interviews consisted of a bank of images and an entomological box. From the 15 species recorded in the conventional survey, were selected the 10 species (7 pest insects and 3 predatory insects) that showed the highest faunal indices. The images were taken from the wild or in the laboratory by a digital camera, edited in Photoshop and printed on glossy photo paper 180 g in size 10 × 15. The photos of insects smaller than 2 mm (*B. tabaci*, *A. gossypii*, *Planococcus* sp.) Have been enlarged by 15 times. Others, such as *Lagria villosa*, *Diabrotica speciosa*, *Leptoglossus zonatus*, *Cycloneda sanguinea*, *Hippodamia convergens*, *Eriopis connexa* and *Liriomyza* sp. were enlarged by 10 times, while *Gryllus assimilis* was presented in natural size. The entomological box contained the insects pinned by conventional methods of insects assembly, excepting the species *B. tabaci*, *A. gossypii* and *Planococcus* sp. Due to their small size, they were presented in clear plastic bags containing okra branches with the infestation of their plague.

The image and assembled insect of the genus *Liriomyza* sp. were included as controls to check the consistency of data on the local knowledge of the insects studied. This insect was not collected in this survey, but was expected to occur in the study area because of being the most frequent pest in another survey of the insect fauna associated with okra plants, held in the municipality of Itabaiana in the same State of Sergipe.

### Analysis of data

From the items listed in the ethnographic technique “free list” the cultural consensus between the statements of informants from the consensus factor was analyzed. The ANTHROPAC® computer package was used to determine the values of the saliency indices and average frequency of citation.

With the purpose of verifying possible differences in frequency results in faunal conventional survey and frequency obtained on the free list, Spearman correlation coefficient was applied, using the Software Bioestat 5.0 [31]. In order to compare the richness of species recorded using both methods (conventional sampling and free list) the chi-square test was applied.

In order to check any eventual significant differences in registration of insects between the results obtained by different visual stimuli (picture stock and entomological box), the Cochran test was applied through the Bioestat 5.0 software [31]. This procedure was based on the 10 most representative species of conventional entomological survey plus a control species. A binary matrix was created, in which each insect species recognized in the projective interview from the stock photos or the entomological box was considered present. The ones not recognized or under a divergence in the identification were considered as absent.

Then again, to compare efficiency between the visual stimulus using image and dried specimens (entomological box), a table was produced containing the number of species recognized by the informants (correct), the number of unrecognized species (do not know) and the number species that have been confused with another (not correct).

The data of projective interviews were analyzed qualitatively also. All interviews were recorded, transcribed and categorized, grouping all the answers and exploring aspects that stood out in the speech of respondents.

## Results

### Comparison between free-listing technique and conventional survey

Fifteen species were recorded in the conventional survey and ten categories of insects were cited in the free list, where six species were recorded in common by the two techniques. None of the four species of predatory insects collected in the conventional survey was cited in the free list, showing that this technique was not effective in sampling natural enemies of insect pests. Four insects not sampled in the conventional survey of okra were cited in the free list. Regarding the frequencies of insect pests, the results were in agreement with the two techniques (conventional sampling and free list) (Table 1). This interview step had an average duration of 15–20 min.

**Table 1:** Comparison between the frequencies obtained in the conventional entomological survey with frequency, citation order and insects saliency recorded on the free list by 36 okra producers in the Irrigated Perimeter Jacaré Curituba, Canindé de São Francisco, SE, Northeast Brazil

Scientific name	Local name	Frequency index (conventional survey)	Frequency (%) (free list)	Order of quotation (free list)	Saliency (free list)
*Aphis gossypii*	Pulgão	42.3	87.2	3.09	0.37
*Bemisia tabaci*	Mosca branca	37.7	76.9	2.23	0.52
*Phenacoccus* sp.	Cochonilha	15.2	69.2	1.11	0.67
*Diabrotica speciosa*	Vaquinha	0.16	51.3	4	0.13
*Gryllus assimilis*	Grilo	0.15	15.4	2.33	0.10
--	Lagarta rosca^a^	--	15.4	1.83	0.12
--	Formiga^a^	--	15.4	1.5	0.13
--	Lagarta-verde^a^	--	12.8	2	0.09
*Lagria villosa*	Podador	0.20	5.1	1	0.05
--	Cigarrinha^a^	--	2.6	1	0.02

^a^Especimen not collected in the conventional survey of entomofauna

According to the salience index, the mealybug (*Planococus* sp.) was the species with the highest value (0.67). This insect can thus be considered the most important in the perception of farmers consulted. According to informants, this is the most recent pest to infest okra. Both in the conventional survey and free list, it was the third most common species, showing a 69.2 % citation rate with the latter technique.

The aphid (*A. gossypii*), whitefly (*B. tabaci*) and “vaquinha” (*D. speciosa*) showed similar a situation as the mealybug with respect to salience index (0.377, 0.529 and 0.133 respectively) and citation frequency (87.2, 76.9 and 51.3 %, respectively). Both were among the first being cited. From the information provided by farmers on the free list, it can be inferred that, just as in the conventional survey, the species *Planococus* sp., *A. gossypii*, *B. tabaci*, and *D. speciosa* are key okra pests.

The richness values determined using the two techniques showed no significant differences (*χ*^2^ = 0.66, *p* = 0.54). Comparing the faunistic frequency index of the most representative insects found in the conventional survey with the frequency obtained from the free list, there was a strong association according to Spearman correlation (r = 0.8286, *p* = 0.0415). Thus, there was similarity between the data obtained in conventional sampling and free list sampling.

### Comparison of visual stimuli and dried specimens in checklist-interview technique

The term “pest” was used to name all insects, including natural enemies in the checklist interview, and predatory insects were the least recognized species. Comparing the data obtained by using images with the responses to dried specimens (entomological box ), it was observed that among the 11 species indicated by farmers in the checklist interview, there were 91 acknowledgments by the informants using photographs, and 209 using the entomological box. These differences were significant according to the Chocran test (*χ*^2^ = 118, DF = 1, *p* < 0.0001) (Table 2). Thus, the use of entomological box proved to be a more efficient visual stimulus method than the photographs in the survey of insect fauna. This interview step had an average duration of 40–60 min. Generally, the informants had difficulties in interpreting the images and consequently in identifying the species, probably due to lack of familiarity with the photographic presentation of scale insects, especially when the photographs were enlarged. The species whose photographs were most enlarged (*B. tabaci, A. gossypii* and *Phenacoccus* sp.) were the least recognized in interviews in which images were used as visual stimuli. However, when the informants were shown a transparent plastic bag containing a branch infested with the same insects, they quickly recognized them.

**Table 2:** Species of insects in the most representative survey of the entomofauna used in projective interview with stock photos and entomological box in the Irrigated Perimeter Jacaré Curituba, Canindé de São Francisco, SE, Northeast Brazil

Species	Stock photos			Entomological box		
	Do not know^a^	Correct^b^	Not correct^c^	Do not know^a^	Correct^b^	Not correct^c^
*Bemisia tabaci*	16	9	11	4	31	1
*Aphis gossypii*	20	6	10	3	31	2
*Phenacoccus* sp.	15	11	10	2	32	2
*Lagria villosa*	22	11	3	13	21	2
*Diabrotica speciosa*	17	9	10	6	24	6
*Leptoglossus zonatus*	25	7	4	23	11	2
*Cycloneda sanguinea*	28	3	5	18	12	6
*Hippodamia convergens*	31	3	2	26	9	1
*Eriopis connexa*	31	2	3	30	3	3
*Gryllus assimilis*	6	30	0	1	35	0
*Liriomyza* sp*.* ^d^	26	0	10	28	0	8
Total	237	91	68	154	209	33

^a^Number of non recognized species

^b^Number of recognized species

^c^Number of species confused to others

^d^Control species

The cricket (*G. assimilis*) was the only species recognized by all informants from photographs, probably because it was the only insect whose photograph showed the animal in its natural size. The whitefly (*B. tabaci*) was not recognized from the photograph by any of the informants, but was recognized by 86 % of the informants (31) when we used the entomological box. The informants reported that they recognized the presence of smaller insects such as *B. tabaci*, *A. gossypii* and *Phenacoccus* sp. mainly through the symptoms in okra caused by their attack, so the use of photographs was found to be rather ineffective in the farmers’ recognition of these pests. The control species was not recognized by any of the informants with either of the techniques in question, ensuring the reliability of the information obtained.

## Discussion

### Comparison between free-listing technique and conventional survey

Based on the knowledge of farmers in the free list, as well as in conventional sampling, the species *B. tabaci*, A*. gossypii*, *Phenacoccus* sp. and *L. vilosa* were the key pests of okra. Msoffe et al. [17] compared data from interviews with field survey data for monitoring large mammals in northern Tanzania and found that interviews could provide important information about the presence/absence and distribution of species in large areas. Likewise, interviews were effective for monitoring *Acinonyx jubatus* cheetahs in Kenya [32], beluga whales in Alaska, diet, migration, and reproduction in the bluefish (*Pomatomus saltatrix*) along the Brazilian coast [33] and identification of mammalian carnivore feces in the Reserva Natural del Bosque Mbaracayú (RNBM) in eastern Paraguay [34]. These authors proposed an integrated approach to study and monitor wildlife using primary (field survey) and secondary (interviews) data sources, by involving the knowledge of local people. The present study reinforced this idea, showing that it was possible to perform a rapid survey of the key pests of a crop through ethnographic techniques such as free list.

No predatory insect was cited by the informants on the free list and the term “pest” was used to name all insects, including natural enemies in the checklist-interview. Unlike in the study of Silvano and Begosi [35], in which most of the feeding interactions reported by fishermen agreed with data from the biological literature on fish diet. Costa-Neto and Magalhães [36] and Petiza, et al. [37] found that the term “insect” was a broad semantic category, and that insects were mostly recognized by their negative aspects. This trend points to the need to test other approaches that are able to bring out local knowledge about predatory insects and to develop educational strategies that reinforce the positive aspects relating insects to farmers, such as their importance in biological control of pests and diseases.

The survey of the entomofauna using the free listing technique was performed in a shorter time as compared with the conventional survey or interviews using the checklist technique. Both the free listing and checklist interviews are considered forms of semi-structured interviews (in which questions are partly made by the researcher before going to the field), which have the characteristic of being easy with quick reference when compared with the open-structured interviews or not requiring the sample to include a large number of responders [22], thereby making it the most recommended type of interview for RBA.

Accordingly, Anadón and Ballestar [38] described a quick technique to obtain a predictive model of absolute abundance of animals on a large scale based on data modeling obtained from local ecological knowledge (LEK).

According to Silvano and Begosi [35], studies on fishermen’s LEK may also help to better define sampling designs for future biological fisheries surveys with reliability and accuracy, to improve biological knowledge and management practices, specially (but not only) when formal biological studies are scarce.

Non-sampled insects in the conventional survey of okra were cited in the free list, probably because they cause accidents in the field (ant sting) or because they attack other important crops such as corn and beans (this seems to be the case of the corn borer and leafhopper). In Baniwa ethnic group in the state of Amazonas, northern Brazil, Petiza et al. [37] reported that the most important insects for the Indians were those that caused some sort of accident (sting, burn, bite) or representing some kind of harm for humans. Specifically in the semiarid region of Brazil, where the present research was conducted, there is a paucity of studies on local forms of human-insect interaction in peasant or agricultural societies.

### Comparison of visual stimuli and dried specimens in checklist-interview technique

With respect to the visual stimuli used in previous studies, the use of photographs is quite controversial, appearing to be ineffective in some cases and highly effective in others. Thomas et al. [21] and Santos et al. [39] reported that the use of photographs was more effective than the use of dried specimens of plants by their informants. Similarly, photographs were effective in recognizing snakes by students of a basic education agricultural school located in the semiarid region of northeastern Brazil [40], and in four studies, we analyzed fishermen’s LEK about the fishes along the coast of Brazil and Australia [35–41]. In contrast, Monteiro et al. [42] found that the use of pictures was not satisfactory, where less than 5 % of medicinal plants analyzed were recognized by informants in the preliminary phase of the research, in a semiarid region of northeastern Brazil. In other ethnobotanical studies, informants showed difficulties in recognizing plants from photographs [25–43].

Compared to insect pests, the size factor (in the images) and the symptom of damage caused in the crop were decisive for the recognition of the insect. Informants reported that they recognized the pests *B. tabaci*, A*. gossypii* and *Phenacoccus* sp. mainly through the symptoms caused by the attack of these insects in okra. Sometimes some informants spent more than a minute analyzing a particular photograph, showing difficulty knowing about the insect in the illustration. But when seeing the mounted insect (or clear plastic bag containing the branch infested with these pests), they were immediately able to give the popular name and to provide characteristic information about the pest.

A similar result was observed among the Baniwa Indians, in which the features most used to identify insects were size and color. [37] For reliable identification of insects, we recommend the use of photographs featuring the bug in its natural size, or the mounted specimen, and symptoms of damage. In this regard, ethnoentomological studies have advantages over research on other taxonomic groups of animals, where dried specimens can be easily transported in entomological box for identification use in checklist-interviews [26]. Another positive aspect is that there is hardly any loss of color or texture due to mounting and conservation techniques, as with plants [21]. Unlike vertebrates, obtaining dead insect specimens for scientific purposes does not violate laws that protect nature.

Ethnoecological studies may also help in promoting dialogue and cooperation between local people and scientists [10, 12–44]. The present study may reinforce this idea, where this research has contributed to the discussion of an participatory integrated management plan for controlling pests of okra crops.

## Conclusion

This study provides evidences that techniques based on the record and analysis of local knowledge about insects are effective for quick sampling of insect pests, but ineffective in sampling insect predators.

In conclusion, the techniques that evoke local entomological knowledge  do not replace conventional scientific sampling due to the need to identify insect pests and their natural enemies. Notwithstanding, these techniques can be an important complementary tool in decision making on Integrated Pest Management and rapid biodiversity assessment.

It is recommended the use of assembled insects, infested branches or photos of the symptoms of damage caused by pests in projective interviews, since these stimuli more easily maintain the original properties of insects, facilitating the identification of species by the informants.
